# The Synergistic Effect of Ester-Ether Copolymerization Thixo-Tropic Superplasticizer and Nano-Clay on the Buildability of 3D Printable Cementitious Materials

**DOI:** 10.3390/ma14164622

**Published:** 2021-08-17

**Authors:** Yu Wang, Yaqing Jiang, Tinghong Pan, Kangting Yin

**Affiliations:** College of Mechanics and Materials, Hohai University, Nanjing 211100, China; wangyuhhu@hhu.edu.cn (Y.W.); thpan@hhu.edu.cn (T.P.); ktyin@hhu.edu.cn (K.Y.)

**Keywords:** 3D printing, cementitious materials, buildability, rheology

## Abstract

The shape retention ability of materials deposited layer by layer is called buildability, which is an indispensable performance parameter for successful 3D printable cementitious materials (3DPC). This study investigated the synergistic effect of nano-clay (NC) and thixotropic superplasticizer (TP) on the buildability of 3DPC. The rheological parameters and static yield stress are characterized by the rheology testing, the green strength is measured by a self-made pressure tester, and the fluidity is tested by flow table. Results indicate that NC significantly increases the growth rate of static yield stress and green strength and TP can improve the initial rheological parameters and fluidity, which ensures the initial stiffness and workability of printed materials. The mixture with 7‰ (by mass of cementitious materials) NC and 3‰ TP obtains excellent extrudability and buildability, due to the synergistic effect of NC and TP. Based on the rheology testing and specific printing experiments, a printable window with 1.0 Pa/s~2.0 Pa/s of the rate of static yield stress evolution over time (RST) or 170 mm~200 mm of fluidity is established. This work provides theorical support for the control and evaluation of rheological properties in 3DPC.

## 1. Introduction

3D printing of cementitious materials is a novel construction technique, which possesses advantages of higher construction efficiency, formwork free, intelligence and so on. In traditional casting techniques, concrete products are formed in molds or subtracted by milling, while 3D printing of cementitious materials is an additive manufacturing approach to build 3D printed constructs through layer deposition and stacking so that the automated process can be finished from digital models [1,2,3,4]. During the accumulation of 3D printable cementitious materials (3DPC) layer by layer, the first layer needs to support its own weight and later the successive deposited layers by depending on the static yield stress which changes with time. The ability of printed objects to retain their geometry is called “buildability” [5]. To maintain the structural stability of extrusion-based 3DPC, it is critical to design a “printable ink” with proper rheological properties that guarantee pumping, extrusion and subsequent rapid standing behavior of 3DPC [6,7,8,9].

The buildability of 3DPC is related to the capacity of printing several continuous layers, i.e., the materials should have suitable fluidity to avoid the fracture of printed filaments due to the inability to be continuously extruded [10,11]. Secondly, the layers need to bond tightly together when being stacked so that the appearance of cold joints and any reduction of the printed objects’ strength can be avoided [12]. Finally, the materials should have sufficient initial rigidity to ensure the initial shape retention capability after the extrusion from the nozzle. Otherwise, the collapse or tilt due to the weight of printed layers and later deposited layers may cause damage to the printed structural components [13]. Le et al. investigated the numbers of buildable layers to quantify the buildability of fresh 3DPC on the basis of the stacked lower layers without observable deformation [14]. Based on the assumption that the growth of static yield stress has a linear relationship with time during the hydration induction period, Yuan et al. [15] improved the thixotropy of fresh mortar and established the linear equation slope to evaluate and predict the 3DPC buildability. Casagrande et al. [16] performed uniaxial compressive tests on cylindrical samples by varying the testing procedures to evaluate the buildability of 3DPC and predict critical number of layers. Zhang et al. [9] assessed the buildability of printed objects by recognizing the visible deformation or collapse. More and more studies have been conducted on the buildability of 3DPC. However, technologies on 3DPC buildability performance adjustment have not reached full maturity so far, and in addition, the methods for evaluation of concrete performance at home or abroad are not unified.

The rheology of the deposited materials plays an important role in studying the buildability of 3DPC [17]. Meanwhile, the yield stress is a significant rheological parameter for representing the buildability of 3DPC [17,18]. The main rheological requirements of successful structural building are the range and type of yield stress [19]. Among all the rheological characteristics, the shape stability of the fresh 3DPC can also be described by its thixotropic behavior [20]. The addition of nano-clay is a common method to improve the thixotropy of materials. The different types, dispersion media and exfoliation level of nano-clays can change the thixotropic properties and stacking ability of extruded 3DPC [21]. As a means of nano-scale reinforcement, Papatzani et al. investigated the pozzolanic properties of organomodified, inorganic and exfoliated nano-montmorillonite dispersions seeking additional nucleation sites in cement paste [22,23,24]. Yu et al. reported that organomodified montmorillonite improved the damping capacities of cement paste better than montmorillonite [25]. Panda et al. improved the yield stress and early strength of fresh mixtures and obtained higher buildability with minimum deformation by adding small amounts of nano-clay [26]. Mendoza Reales et al. [27] studied the effects of nano-silica particles on the fresh state properties of 3DPC by comparing nano-silica with micro-silica, metakaolin and nano-clay. For computing ideal printing parameters (such as layer height, time for extruding one layer, horizontal velocity and so on), the rate of thixotropic build-up is obtained through the rheological testing results. Through a novel thixotropy model, van den Heever et al. showed that SiC nanoparticles improved the 3D printing buildability by enhancing the thixotropy [28]. Meanwhile, the addition of superplasticizer and materials proportion optimization are also effective ways to control the rheology of materials. To meet the requirements of an ideal 3DPC, Manikandan et al. [29] added silica fume and superplasticizer in fresh paste to control the rheological properties. Through optimizing the aggregates and binders, Papachristoforou et al. [30] demonstrated that in the printable range, which was found to be between 180–240 mm (flow table values), a maximum workability time of 30 min was obtained without using any retarder additives. In order to achieve ideal buildability of 3DPC, many works on obtaining suitable rheology have been carried out. However, the synergistic effect of nano-clay and superplasticizer on buildability of 3DPC has not been studied in depth yet.

In this study, the synergistic effects of nano materials and superplasticizer are explored to improve the structural build-up ability of 3DPC products. Nano-clay, polycarboxylate superplasticizers (PCE) and a self-made ester-ether copolymerization thixotropic superplasticizer (TP) were used to investigate the rheological properties of 3DPC buildability. Rheological properties tests have been conducted for characterizing the rheological parameters and static yield stress and the green strength is tested by a self-made pressure tester, while the fluidity value is obtained by flow table tests. Moreover, a rheology model for parameter analysis and evaluation methods to assess the buildability of 3DPC are proposed and the buildability verification of mixtures has also been conducted. This work provides theorical support for the control and evaluation of the effect of rheological properties on the buildability of 3DPC.

## 2. Materials and Methodology

### 2.1. Materials and Mix Proportion

**Figure 1:**
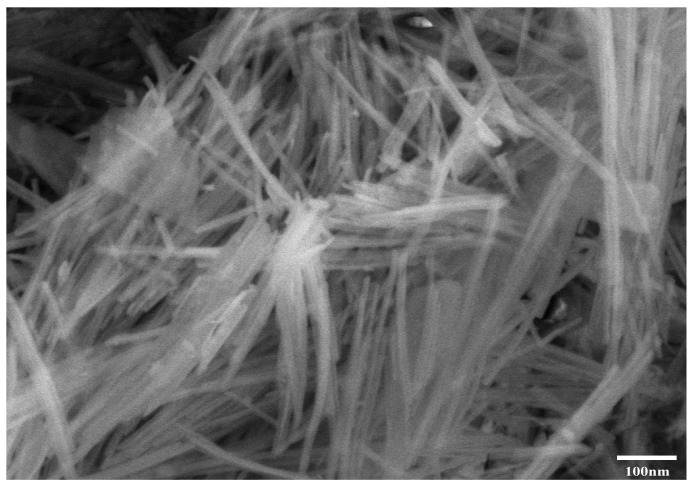
SEM of NC.

Portland cement (Type II, 42.5 grade, Conch Cement Co. Ltd., Huai’an, China) and tap-water were used in the mixtures. A commercial highly purified and activated nano attapulgite clay (Huai’an Research Institute of Hohai University, Huai’an, China) was used for thixotropic admixture. The chemical compositions and Blaine fineness of the cement and nano-clay (NC) are given in Table 1. The NC forms needle-like particles with 350 nm in average length and 40 nm in diameter. A scanning electron microscope (SEM) image of the NC is shown in Figure 1. Commercial river sand is used as fine aggregate, with fineness modulus of 2.2 and maximum size of 1 mm.

**Figure 2:**
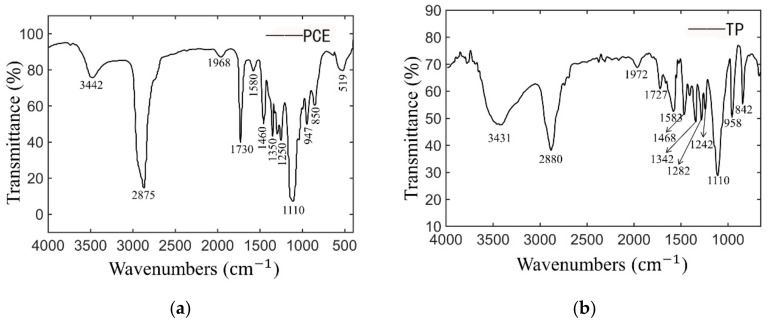
IR spectra for (**a**) PCE and (**b**) TP. (3442 cm^−1^ and 3431 cm^−1^ belong to the stretching vibration peak of hydroxyl group. A strong characteristic absorption band correlating with the deform vibration of (–C–H) group was observed at 2875 cm^−1^ and 2880 cm^−1^. The absorption peaks at 1727 cm^−1^ and 1730 cm^−1^ can be attributed to the stretching vibration of carbonyl (–C=O) group in the ester (–O–C=O) group. The absorption peaks at 1580 cm^−1^ and 1583 cm^−1^ can be attributed to the stretching vibration of carboxylic group (-COOH) group.).

Two kinds of superplasticizer are used in this study: a self-made ester-ether copoly-merization thixotropic superplasticizer (TP) and a commercial polycarboxylate superplasticizer (PCE) purchased from Sobute New Materials Co. Ltd. (Nanjing, China). The TP was synthesized as follows: the polymerization monomers of TP are prepared with sodium allyl sulfonate monomer (SAS), acrylic acid monomer (AA), macromonomer polyoxyethylene unsaturated ester (MPEG400MA) and α-methacryl-ω-hydroxy poly (ethylene glycol) ether (HPEG2400, molecular weight is about 2400). A molar ratio (AA:SAS:MPEG400MA:HPEG2400 = 3:1:0.3:0.7) is determined, and ammonium persulfate (4% of the total mass of polymerization monomer) is as the TP initiator. MPEG400MA, HPEG2400, SAS and water are fed into a three-necked flask at one time. AA and ammonium persulfate are diluted to 20% concentration solution, respectively, and they are dripped in every 20 min. The synthesis is carried out at 80 °C and the dripping is completed within 2 h, then the temperature is maintained for 4 h. After cooling the solution pH is adjusted to 7 using 40% NaOH. Finally, an ester-ether copolymer thixotropic superplasticizer (TP) with 44.3% solid content is obtained. The FTIR spectra of the TP polymer and PCE polymer is recorded by using a Nicolet iN10 instrument (Thermo Fisher Scientific Co. Ltd., Waltham, MA, USA). The 1~2 mg of purified TP or PCE sample is ground with 200 mg KBr, and embedded in a mold to be pressed into a sheet. Each sample is tested between 400 cm^−1^~4000 cm^−1^ wavenumber and scanned 32 times at the resolution of 4 cm^−1^. The infrared spectrograms of TP and PCE are shown in Figure 2.

The gel permeation chromatograph (GPC) results of TP and PCE have been obtained in a previous group work and are briefly presented here [31]. The weight-average molecular weight and number-average molecular weights of TP polymer are 46,960 g/mol and 23,863 g/mol, respectively. The weight-average molecular weight and number-average molecular weights of PC polymer are 38,993 g/mol and 19,930 g/mol, respectively.

Ten proportions of ingredients are shown in Table 2. The water to cement ratio (w/c) of mixtures is 0.32 by mass, and the fine aggregate to cement ratio is 1.2:1 by mass. The mixture with 3‰ addition of PCE is used as the control mix. Single-doped TP addition is controlled from 2.5‰~3.5‰ in cement for adjusting the slump-retaining and flowability. NC addition in cement varies from 7‰, 8‰, 9‰ for the verification of its thixotropic and rigid strengthen property. Mixtures with fixed TP content of 3‰ and NC dosage ranging from 7‰~9‰ are aimed at improving the workability as well as the thixotropy and strength of 3DPC buildability.

### 2.2. Mixing Protocols

An NRJ-411A planetary mortar mixer (Wuxi Jianyi Experiment instrument Co. Ltd., Wuxi, China) is used for mixing the cement mortar in this study. The mixing protocols are as follows: Firstly, after being weighed, all the dry mixture ingredients, such as cement, NC and sand are poured into the mortar mixer for mixing homogenously for 1 min. Secondly, water with TP is slowly added into the dry powder for mixing for 1 min at low speed. Next, the process is stopped for 30 s and the cement mortar adhered on the edge of mixer pot is scraped off with a shovel. Finally, the mixture is subjected to mixing at a fast rate for 90 s. The stirred mortar was placed into a suitable container for performing the rheological tests, fluidity tests and green strength tests immediately.

### 2.3. Rheological Properties Test

**Figure 3:**
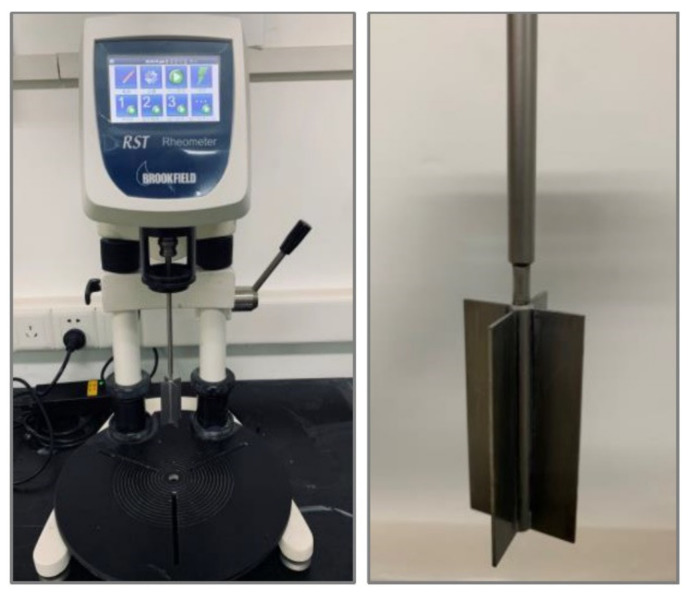
Rheological properties test of cement mortar.

A commercial rheometer (RST-SST rheometer, BROOKFIELD, Middleborough, MA, USA, shown in Figure 3) is used to measure the rheological properties of the fresh mortars with different mix proportions in this study. The fresh cement mortars are poured into a barrel with a diameter of 70 mm and a height of 100 mm. The rheometer rotor is a four-blade vane that 15 mm in diameter and 30 mm in height. For the purpose of monitoring and recording the shear stress and shear rate constantly, the rotor is rotated to generate the simple shearing flow. Then, the testing data is fitted in the relevant rheological models for the required parameters. Three samples are tested to determine an average value for the same batch.

#### 2.3.1. Hysteresis Loop Test Protocol

**Figure 4:**
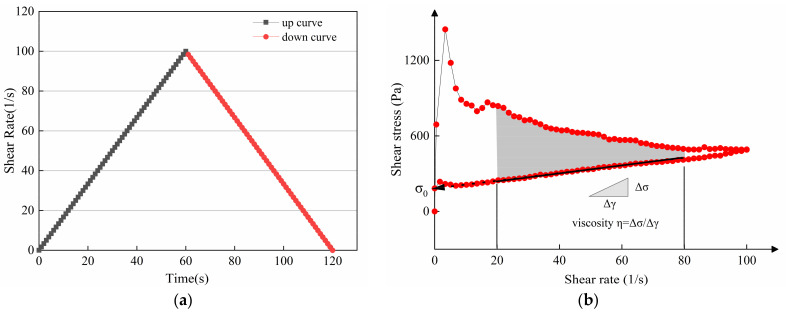
(**a**) The rheology test protocol and (**b**) the hysteresis loop obtained by the test.

The hysteresis loop test is a popular way to quantify the thixotropy of cement mortar, as shown in Figure 4a. Thixotropy is associated with the energy that forms and breaks the flocculation of cement particles [32,33]. The enclosed area shown in Figure 4b (usually called thixotropic loop), can be expressed by Equation (1):

(1) $$A=\int_{\gamma_{min}}^{\gamma_{max}}\tau_{1}\gamma d\gamma -\int_{\gamma_{min}}^{\gamma_{max}}\tau_{2}\gamma d\gamma ,$$

where *A* represents the calculated results of thixotropic area, γ represents shear rate (1/s), $\tau_{1}$ and $\tau_{2}$ represents the high and low shear stress, respectively.

#### 2.3.2. Static Yield Stress Test Program

**Figure 5:**
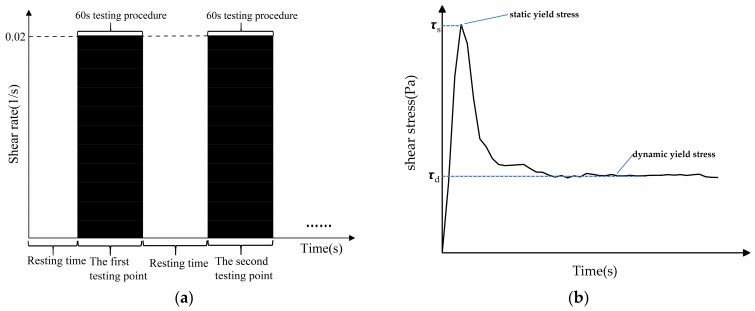
(**a**) The rheological test procedure for time-dependent static yield stress is conducted after the resting time (usually after the mixing time) and (**b**) the static yield stress is obtained in the test.

The time-dependent growth of static yield stress is measured quantitatively in the testing program as shown in Figure 5a. The fresh cement mortar is divided into 10 groups for testing static yield stress at 10 resting times. All the samples are prepared after the mixing procedure before conducting the shearing test, which begins at 0 s time. For the first eight resting times, the static yield stress is tested every 150 s, while the last two groups are tested at resting times of 2000 s and 3000 s, separately. The constant shearing rate is set at 0.02/s and the measured maximum shearing stress in the Figure 5b is defined as static yield stress, which can be used to characterize the build-up ability during the early hydration stage of 3DPC.

### 2.4. Fluidity Test

**Figure 6:**
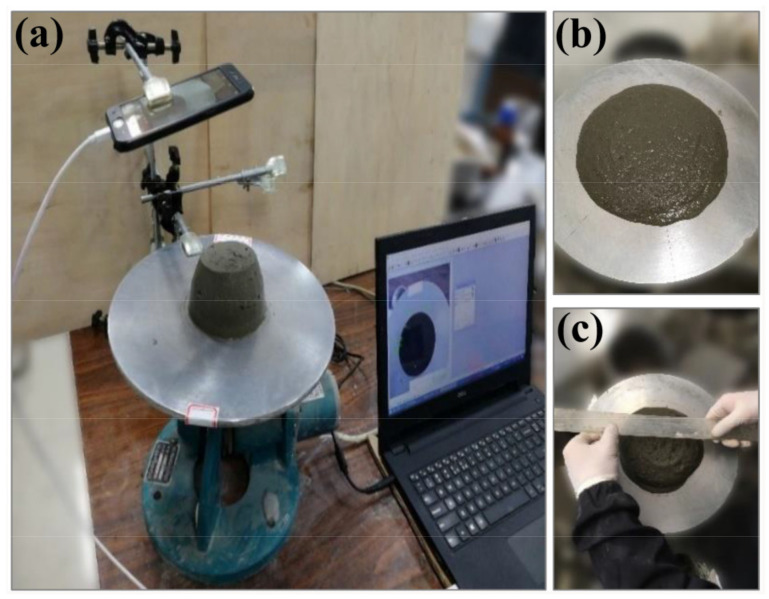
The fluidity test system: (**a**) the shooting and image transmission system, (**b**) the sample on the flow table and (**c**) the measurement of fluidity value.

The flowability value of 3DPC is obtained by performing the fluidity test based on ASTM C1437-15 [34]. In order to enhance the efficiency of data collection, a shooting and image transmission system and a reference coordinate is established on the flow table, as shown in Figure 6. During the test, the average diameter is calculated through the image system by using image processing software. Three mixed samples of the same batch were tested at a certain time for observing how the fluidity changed over time. The fluidity test of cement mortar is used for evaluating the flowability of 3D printing cement-based materials. The results are compared with the rheological parameters and analyzed for the correlation between it and different rheological results so that the flocculation rate and structuration rate can be better observed.

### 2.5. Green Strength Test

**Figure 7:**
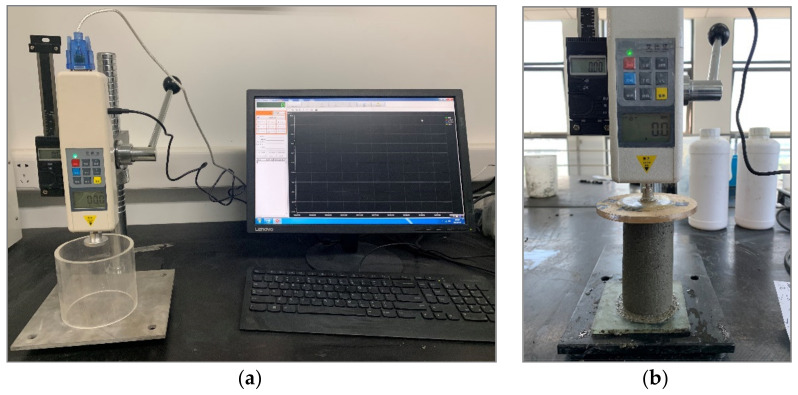
(**a**) The green strength test instrument for testing and recording and (**b**) the sample under test.

The cement mortar is stirred with the same method described in Section 2.3.1. After mixing, the cement mortar is poured into a cylinder plastic mold (with 50 mm in diameter and 100 mm in length) in three layers. The sample is packed tightly by using a tamping rod for tamping 15 times after each layer is added. Then, it is demolded at a fixed time and placed in a self-made pressure testing device, as shown in Figure 7. A progressively increased load is applied to the specimens until vertical displacement of the sample height reached 15%. The ratio of measured load to the bottom area of the cylindrical specimen was converted to green strength. In this study, the green strength is obtained on three samples of the same batch at certain time intervals (0, 150, 300, 450, 600, 750, 900, 1050, 2000 and 3000 s, respectively).

### 2.6. Buildability Verification

**Figure 8:**
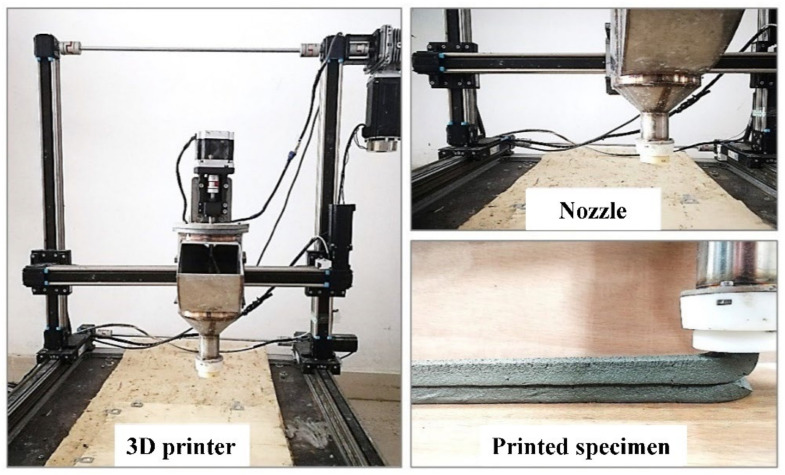
Medium sized gantry 3D printer.

As shown in Figure 8, a 3D printer with a forming space of 500 mm × 500 mm × 500 mm is used in this study. The nozzle is 20 mm in diameter. The printed material is transported to the barrel through a pipeline and extruded at the speed of 60 s/layer with a pre-mixing procedure before extrusion. The printing time of each layer is about 300 s after the cement is mixed with water. The samples with all the proportions are subjected to 3D printing tests in this study to evaluate the buildability of the materials according to the actual printed state.

### 2.7. TOC Adsorption Measurement

A total organic carbon analyzer (TOC multi 2100, Jena Analytical Instrument Co. Ltd., Jena, Germany) is used in this work. Thirty grams of TP solutions with different concentrations (1, 2, 3, 4 and 5 g/L, respectively) are prepared and 5 g of cement powder is mixed with the solutions for 5 min. After standing for 5 min, then samples are filtered and separated for centrifuging for 20 min at 8000 rpm. The upper suspension of the TP solution is filtered through a membrane with 0.22 μm in pore diameter for TOC measurements. The testing range is from 4 ppb to 4000 ppm. The TOC value is obtained from the subtraction of inorganic carbon (IC) from the total carbon (TC). The adsorption amount of TP is calculated by the solution concentration difference before and after the adsorption.

### 2.8. Zeta Potential

A Nano ZS90 Zetasizer analyzer manufactured by Malvern Panalytical Co. Ltd. (Shanghai, China) is used in this work. The zeta potential on the surface of cement particles in the cement suspension with a certain concentration of TP is measured. The mass ratios of PCE or TP to cement were 2.5‰, 3‰ and 3.5‰. The samples are diluted with deionized water up to 1000 times for mixing and ultrasonic dispersion. The supernatant of the cement suspensions is injected into the samples for recording the stabilized zeta potential values.

## 3. Results and Discussion

### 3.1. Flowability

#### 3.1.1. Rheological Parameters

Rheological parameters are critical to the workability of fresh 3DPC. Corresponding to the initial fluidity, the initial rheological properties reflect the primary flowability of printed cement-based materials. Although excellent thixotropic properties are essential for 3DPC, the initial stiffness of printed materials also has great influence on their buildability [28]. 3DPC requires a sufficient yield stress growth rate to be compatible with the vertical building rate. The yield stress development over time is called the structural accumulation. The method for accelerating the structural accumulation is to add the admixtures that can increase the initial yield stress or raise the thixotropy, which is capable of enhancing yield stress in a short time. Therefore, the early strength of materials can be obtained indirectly from the measurement of yield stress.

**Figure 9:**
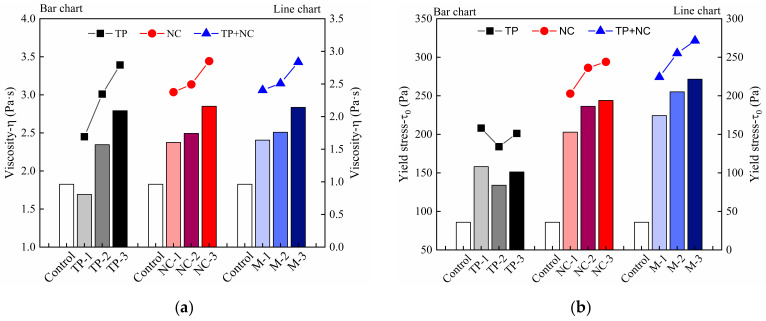
The initial rheological parameters: (**a**) viscosity, (**b**) yield stress of 3DPC that added TP, NC and PCE.

The test method described in Section 2.3.1 is used on the fresh mortar. The down curve of hysteresis loop is fitted to determine the yield stress and viscosity. As can be observed in Figure 9, the initial yield stress and viscosity showed a significant increase in the samples with NC, regardless of whether single-doped NC or double-doped NC and TP were added together into the cement mortar. This may be attributed to the small particle size of NC [18]. Acting as a nano-filler, NC filled the gaps between cement particles and agglomerates, so that more touchpoints can form an interlocking microstructure effectively, which increases the initial viscosity [18]. On the other hand, the nano size enables clay particles to multiply the specific surface area and increase the nucleation sites of cement mortar [22,23]. Thus, they accelerate the cement hydration, densify the cement mortar structure and this results in the improvement of the initial yield stress of samples [24,27]. In addition, by comparing the initial viscosity and yield stress of the samples added 3‰ PCE to that added 3‰ TP, it is evident that the enhancement effect of TP is more obvious than that of PCE, but worse than that of NC.

**Figure 10:**
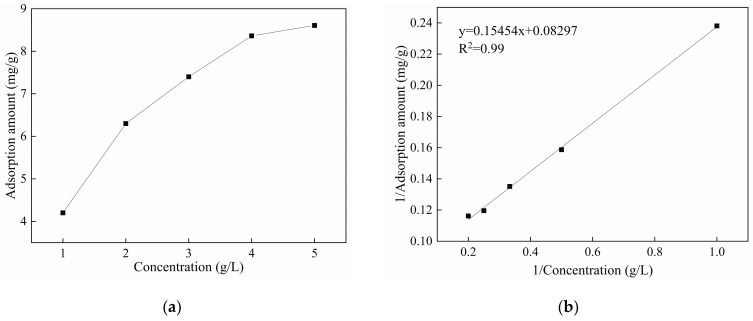
(**a**) The relationship between adsorption amount of TP on cement surface (in mg per g of cement) and TP concentration, and (**b**) the model of Langmuir adsorption isotherms.

From the results illustrated in Figure 9, the initial viscosity and yield stress in the samples with single-doped TP are higher than those of control mix. With more TP content, the initial viscosity increased continuously. However, once the TP dosage is over 3‰, the initial yield stress of samples decreased slightly with comparison of the samples with a TP content of less than 3‰. It can thus be inferred that 3‰ TP dosage may be a turning point in optimizing materials rheological properties. For verifying this, the adsorption amount results of TP were obtained and are plotted in Figure 10. It shows that the adsorption amount of TP on the cement particle surface increased with TP concentration. The adsorption rate of TP is high at low concentrations, but it tends to slow down when the TP concertation reaches 4 g/L (2.89‰ TP addition in cement mortar) and an equilibrium value of the amount of adsorption appears. According to Figure 10, the calculated saturation adsorption amount of TP on cement particles is 12.05 mg/g on the basis of the Langmuir adsorption formula [35]. The TP molecules are adsorbed quickly due to the high surface activity of cement particles at an early age [36]. As the occupation of the active sites on the surface progresses, the adsorption rate of TP slows down and the adsorption amount tends to an equilibrium value, which is called the saturation adsorption amount [37]. It can be inferred that the dosage of 3‰ TP, at the turning point of this equilibrium value, is an optimal dosage to realize the effective utilization of TP.

Compared with the control mix in Figure 9b, cement-based materials mixed with TP and NC depict a significant yield stress increase for more addition, especially in the mortar with double-doped admixtures, where the value increased more than double that of control mix. By fixing the TP content at 3‰ and adding NC dosages from 7‰ to 9‰ to the mortar, the increase of viscosity is close to that of materials added TP or NC alone since the fibrous structure of NC enables it to be alternately distributed in cement-based materials and form a disordered grid, thereby forming a stable flocculation structure within the material [38]. It is evident that the initial viscosity of printed cement-based materials would not be damaged by changing TP content. In addition, the initial yield stress and the buildability of 3DPC can be improved by TP.

#### 3.1.2. Fluidity Tests

Like the initial rheological parameters of fresh mortar, the initial fluidity also reflecs the initial flowability of printed mortar. Through the regular vibration of a flow table, the state of the cement mortar in the test corresponding to the destruction and rebuilding of microstructure when the dynamic flow action and static stable state of materials occurs alternately during the printing process [39]. The charge state of particles in the suspension significantly affects the dispersion of particles [40]. Superplasticizers are generally anionic surfactants. They are adsorbed on the surface of cement particles, where an electrostatic repulsion is generated by these particles with negative charge and then the cement particles are dispersed in the cement mortar [41]. Hence by evaluating the flow performance of 3DPC by a fluidity test and comparing it to zeta potential of cement particles surface, the measured rheological parameters for correlation analysis can be used for evaluating the flocculation and structuration process better.

**Figure 11:**
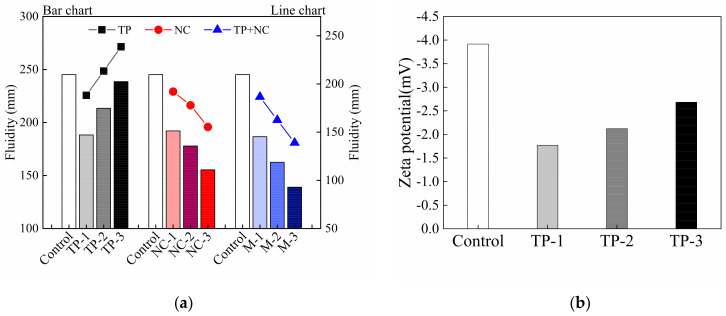
(**a**) The fluidity of mixtures and (**b**) zeta potential of cement particles with TP and PCE addition.

The effect of single blending and compound blending of TP and NC on the initial fluidity of cement-based printed materials is shown in Figure 11a. It is observed that the initial fluidity of samples with TP or NC is lower than that of control mix with 3‰ PCE addition, which indicates that PCE has a good water-reducing and dispersing effect. This is consistent with the zeta potential results, as shown in Figure 11b. It indicates that the cement with PCE has a higher absolute value of zeta potential and causes a better dispersion of cement particles. High fluidity is convenient for pumping and extrusion of printed materials through the pipeline and nozzle, but is not conducive to the “standability” of the materials after being extruded. Combining the fluidity value with the initial yield stress test results of printed materials, it can be found that the addition of TP and NC reduces the fluidity of the material, but greatly increases the initial yield stress of cementitious materials. This shows that both of TP and NC have a positive effect on the buildability of the printed mortar. In the samples added TP only, when the dosage was increased from 2.5‰ to 3‰, the fluidity value increased gradually close to that of control mix, whose fluidity is 245 mm. This shows that TP addition not only has better slump-retaining effect than PCE, but also has good water reducing and dispersion effects on cement mortar. It is also consistent with the zeta potential results in Figure 11b. The absolute values of zeta potential in samples increase continuously with TP addition, which shows electrostatic repulsion effect of TP strengthened the stable dispersion of cement particles thus increased the fluidity of cement mortar. In addition, the flowability of mortar decreases with the increasing addition of NC in the groups with NC. Especially when the fixed TP addition is 3‰, comparing to the control mix with 3‰ PCE, the fluidity loss increases from 24% to 43.2% as the NC content increases from 7‰ to 9‰. Connected with the rheological parameter results, the filling effect and nucleation effect of nanoparticles indicate that appropriate addition of NC is beneficial to the construction performance of printed materials [42]. For 3DPC, there is a contradictory requirement between the extrudability and buildability of materials. During the pumping and extrusion process, the materials must not only have sufficient flow properties to ensure a smooth delivery and extrusion from the nozzle, but also sufficient green strength immediately after extrusion to support its own weight without too much deformation or collapse. As a result, combining the water-reducing, dispersion and slump retaining effects of TP with the advantages of NC can ensure both a smooth extrusion and good support of the printed structure.

### 3.2. Rheology Model for Parameters Analysis

**Figure 12:**
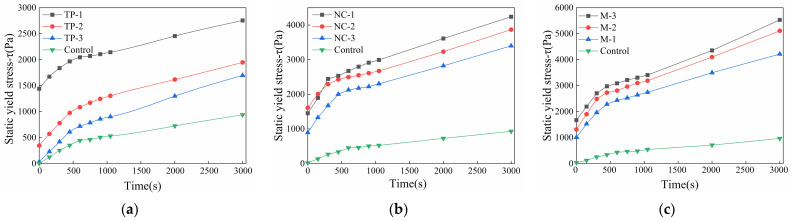
The static yield stress development with time in printed materials with (**a**) single-doped TP, (**b**) doped NC and 3‰ PCE and (**c**) double-doped TP and NC.

By testing the static yield stress of 3DPC over time, the evolution law of printed mortar flocculation structure changes with time can be observed, which is different from the literature [16] on the assumption that static yield stress is linear with time during the induction period of cement hydration. In 3DPC, static yield stress shows a nonlinear increase with time. From Figure 12, it can be seen that the growth of mortar flocculation structure can be divided into two stages: (Ⅰ) a fast rising period, once cement particles mixed with water, cement particles contact with each other rapidly and static yield stress rises quickly until the development rate slows down, and (Ⅱ), a slow rising period, during which cement hydration is dominant and hydration products deposit on the cementitious particles surface continuously. The slow growth of static yield stress manifests that the hydration products increased the total surface area of cement particles and binding force between particles [20]. It can be found in Figure 12a that the growth rate of static yield stress in 3DPC with TP single addition is higher than that of a control mix with 3‰ PCE and the value decreases with higher TP addition. The slump-retaining effect of TP polymer is due to the high density of carboxylic acid (-COOH) groups in the TP polymer, as shown in Figure 2b. The -COOH groups not only have good water-reducing effects, but also can form complexes with calcium ions so as to reduce the concentration of calcium ions and delay the formation of calcium hydroxide crystals for retaining slump [37]. The higher TP addition, the lower the ratio of large particles to agglomerates [43] in 3DPC. It reduces green strength of freshly printed materials.

Figure 12b shows that NC increases the static yield stress and physical flocculation rate of fresh mortar significantly, which is of great importance in improving the 3DPC structure accumulation rate. On the one hand, NC changes the flocculation state of the cement particles, while on the other hand, nanoparticles promote the nucleation of hydrated calcium silicate colloid during the cement structuring process [44]. According to the thermodynamics of cement hydration, a complex fractional and negative exponential correlation between cement hydration and time occurs, instead of a linear relationship. The static yield stress development with time reflects both the flocculation and structuration processes of cement hydration paste. Excessive addition of NC will significantly shorten the flocculation time of cement particles, which will result in a rapid decline in the shaping ability and flowability. As a result, an appropriate addition of NC is extremely important for successful 3DPC structure construction. It can be seen from Figure 12c that when the TP content is fixed at 3‰ and the NC addition is increased from 7‰ to 9‰, the static yield stress value of cement-based materials is larger than that of single-doped groups. In addition, no matter whether before or after the turning point, the growth rate of static yield stress in materials still increases with the NC addition, which indicates that the compounding-doped of TP and NC can significantly improve the flocculation rate and structuration rate of cement-based materials after added in the water.

In 3DPC, two kinds of yield stress (dynamic and static) are associated with flocculation and structure accumulation of fresh mortar. High static yield stress will be produced due to the undamaged microstructure at rest, but when the cement mortar is disturbed by an external force and begins to flow, the microstructure fractures and this results in a lower shear stress (called dynamic yield stress) [7]. A rapid yield stress transition between dynamic and static is necessary to 3DPC, so excellent thixotropy is conducive to the structure of 3DPC. In addition, the ability of structure accumulation (associated with static yield stress) can provide an assessment of buildability [7]. Therefore, the rate of static yield stress evolution over time (RST) is used as an indicator of 3DPC thixotropy to evaluate the buildability of 3DPC in this study.

**Figure 13:**
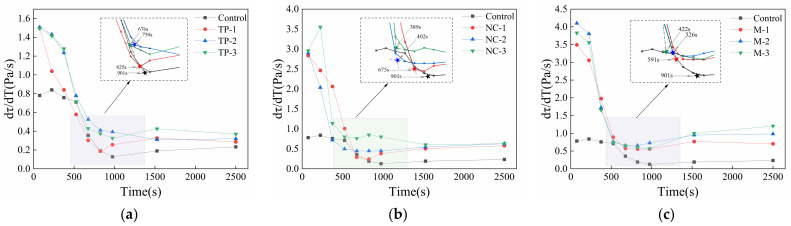
The RST development with time of printed materials with (**a**) single-doped TP, (**b**) doped NC and 3‰ PCE and (**c**) double-doped TP and NC.

The calculation results of RST (the first derivative of static yield stress with time) over time in 10 printed mortar groups are shown in Figure 13. It can be observed that the turning point at which the RST of cement-based materials decreases sharply, meaning the transition point of the materials’ state if changing from physical flocculation to chemical structuration, which represents the duration time of the physical flocculation process and corresponds to the maximum operational time in the mortar [31]. Once the maximum operating time of materials is exceeded, the materials should not continue to be printed, otherwise it will result in interlayer voids that can decrease the durability of specimens [31]. According to the calculation, the 3D printing window times of the 10 groups are shown in Figure 13. There is no doubt that both the addition of NC and TP can promote the rapid flocculation of fresh mortar at early age in all samples, and the nucleation effect of nanoparticles can significantly accelerate cement hydration at an early age. As shown in Figure 13, the RSTs common interval corresponding to the 10 printable window time is 1.0~2.0 Pa/s. In summary, it can be seen that optimizing NC content is an effective method to adjust the rheological properties of 3DPC to meet buildability requirements.

### 3.3. Green Strength Results

During the 3D printing experiments, the bottom layer needs sufficient green strength to bear its own weight and that of the subsequent printed layers. Therefore, there is an inevitable relationship between build-up ability and green strength of fresh mortar, which can be used as an evaluation method for characterizing the buildability. 3D printing is a dynamic phenomenon, hence a printable window time must be established by studying the properties over time of printed materials. Based on rheology theory, this study is aimed at realizing the printability of Portland cement-based 3D printing materials that meets the intelligent manufacturing requirements of extrudability and buildability and establishing the technical index system and evaluation method for supporting engineering applications.

**Figure 14:**
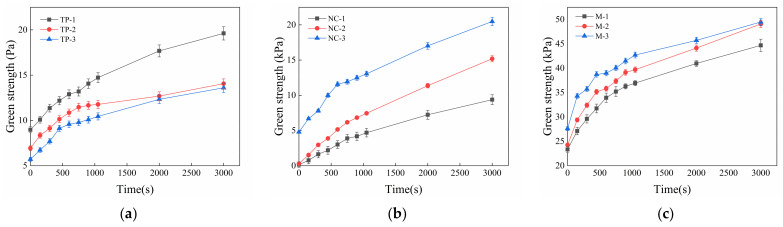
The green strength development with time of printed materials with (**a**) single-doped TP, (**b**) doped NC and 3‰ PCE and (**c**) double-doped TP and NC.

Figure 14 shows the green strength development over time in printed materials with single-doped TP, NC and compound-doped TP and NC on the basis of the control group with 3‰ PCE. It is found that the benchmark mortar collapses without additional load after demolding and the green strength developed slowly over time, thus, it is not able to meet the construction accumulation requirements of printed mortar after being extruded from the nozzle, while the addition of TP and NC significantly increased the green strength of materials. It can be observed that the development of green strength over time (similar to the static yield stress development) can also be divided into two steps: a rapid rising period and a slow rising period. From the beginning to the turning point, the strength grows fast over time, while the time-varying rate of strength decreases significantly after the breaking point. As shown in Figure 14a, the plastic strength of mortar decreases 6.4~11.8 Pa and the initial strength decreases 36.4%, when the TP content increases from 2.5‰ to 3.5‰, which can also be used to explain the fact that fresh mortar with low TP addition can print more layers than materials with high TP content. This corresponds to the sustained-release effect of TP, which continues to reduce the water after the TP molecules is released by the cement particles with addition of TP increasing [7]. The green strength of mortar development when 7‰, 8‰, and 9‰ NC are added to the reference mortar is shown in Figure 14b. It can be seen that the green strength gradually increases by 9.1~21.8 kPa when the NC dosage increases from 7‰ to 9‰. The change is greater than for mortar with TP, which shows that NC addition can improve the mortar green strength better than TP, so the green strength can be improved by adjusting the content of NC for better buildability of 3DPC. It can be observed in Figure 14c that the initial green strength of mortar compound mixed with TP and NC can research 45.8~54.2 kPa, which is much higher than that of groups doped with TP and NC, respectively. This shows that materials mixed with TP and NC strengthen the initial stiffness of mortar, which provides a guarantee for the initial construction performance. Moreover, the green strength growth rate of mortar compound mixed with TP and NC before the breaking point time is significantly higher than that of group with single doped TP and NC. As the NC content increases from 7‰ to 9‰, the breaking point time gradually shortens from 750 s to 600 s in the compound mixtures, which shows that compound addition of TP and NC promotes rapid development of green strength in printed mortar after being extruded.

**Figure 15:**
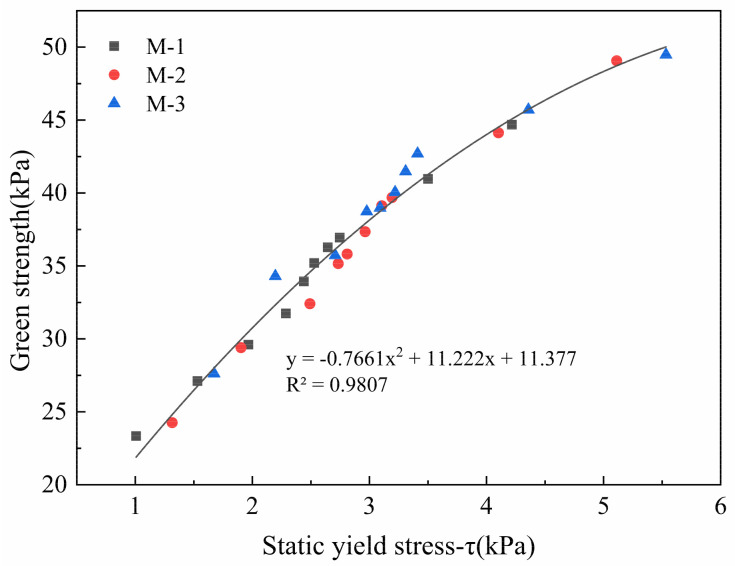
The fitted results of green strength value and static yield stress value.

The test results are fitted in Figure 15 for obtaining the functional relationship between green strength and static yield stress. It is found that R^2^ in the fitted function is 0.98, which indicates that the two have a good correlation. Therefore, according to the fitted results, the static yield stress value can be calculated by testing the green strength of 3DPC. When the rheology test conditions are not available, this provides a simplified test and evaluation method for the early mechanical properties of 3DPC.

### 3.4. Evaluation and Verification of 3DPC

#### 3.4.1. Calculation Model of RST

After Fresh 3DPC being extruded from nozzle, the relationship between initial static yield stress $\tau_{0}$ of printed bottom layer and materials are shown in Equation (2):

(2) $$\tau_{0}\;\geq \;\frac{\rho gh}{\sqrt{3}}$$

where ρ, *g*, *h* represent the density, gravity constant and the height of 3DPC, respectively.

When the printing operation stops, the static yield stress of the bottom layer should be sufficient to support the full weight of the specimen with height of *H_m_*, and the static yield stress *τ*_0_ should meet the requirements of Equation (3):

(3) $$\tau_{0}\;\geq \;\frac{\rho gH_{m}}{\sqrt{3}}$$

It can be seen that RST is a key parameter that affects the 3D printability of cement-based materials. It is determined by the rate of early cement hydration products generation and reflects evolution of 3DPC static yield stress with time. The relationship between RST (*R_thix_*) in fresh mortar and the hydration time is shown in Equation (4):

(4) $$R_{thix}\;=\;\frac{\rho gh}{\sqrt{3}t},$$

After the 3D printing process is determined, i.e., printed layers, printing time interval and so on, Equation (3) can be used to obtain RST that can ensure successful building and meeting the requirement of printing process in the actual printing operation. In this research, the density ρ of 3DPC is 2000 kg/m^3^, the gravity constant *g* is 9.8 N/kg, the height of printed layer is fixed at 0.02 m/layer and the printing time interval is 60 s/layer. To ensure the successful stacking of printed layers without collapse, the minimum static yield stress changing rate in the mortar is calculated as 0.75 Pa/s. As shown in Table 3, comparing the testing value *R_thix_* of each printed layer with the theoretical calculated value *R_thix_*_,_*_min_* in the mixture with 7‰ NC which shows good buildability, it can be found that the materials shows good build-up ability when the measured values are higher than the calculated results. When printing the fifth layer whose printing start time is 540 s, the measured value *R_thix_* is greater than the theoretically calculated value, but *R_thix_* is already less than *R_thix_*_,_*_min_*, which means the tested value is less than theoretical value before printing the sixth layer and the material is uncapable of finishing the printing of the fifth layer. The bottom layer strength of cement-based materials is not sufficient for supporting the subsequent stacked layers weight, so the printing operation should be stopped immediately and the number of printable layers of materials is four, which is consistent with the theoretically calculated number of printable layers. Combined with the initial fluidity window (the range is 170~200 mm) of the printed mortar, it can be found that four mixtures have excellent printability, i.e., TP-1 and NC-1, NC-2 and M-1, whose printable layer numbers are 2, 4, 1, 4, respectively.

#### 3.4.2. Evaluation of 3D Printing Mortar Buildability

The buildability of 3DPC can be obtained by the printable window time combined with the test results from Section 3.1, Section 3.2 and Section 3.3. Fluidity tests are a conventional method to evaluate the fluidity of fresh mortar, in which dynamic shearing and static stable state are alternated during the test so that it can be related with the thixotropy of printed mortar. As a result, the printable windows are defined by the fluidity, initial viscosity, initial yield stress, initial static yield stress and the rate of static yield stress over time. Thus, the printable parameters of mortar with 170~200 mm fluidity, i.e., initial viscosity, initial yield stress, the rate of static yield stress over time and initial static yield stress, are 1.6~2.6 Pa·s, 150~250 Pa, 1.0~2.0 Pa/s, 1.2~1.7 kPa, respectively. This can be used for designing and controlling the extrudability and buildability of 3DPC, based on these windows. Actually, during the printing experiments, to obtain 3D printed cement products that are “printable, standable, and with no cold joint”, controlling the quality of each fresh cement mortar batch and conforming that each batch was printed before the cement hydration induction period starting is an appropriate way.

#### 3.4.3. Buildability Verification of 3D Printing Mortar

**Figure 16:**
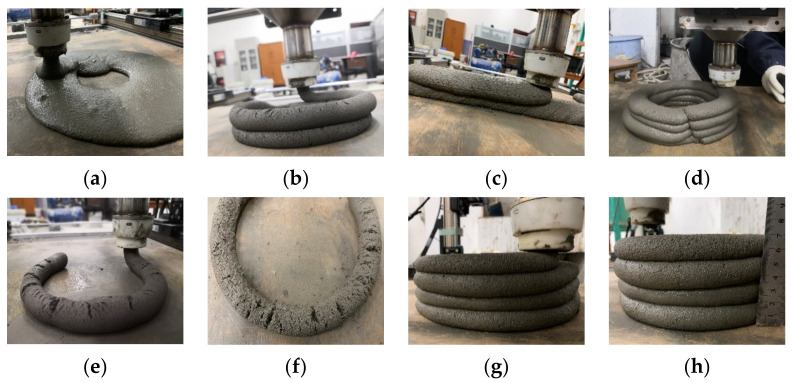
The printed specimens with different dosage of superplasticizer and nano-clay: (**a**) control mix, (**b**) TP-1; (**c**) TP-2, (**d**) NC-1, (**e**) NC-2, (**f**) NC-3, (**g**) M-1 and (**h**) the deformation measurement of M-1 mixture.

Excellent 3D printing buildability requires not only proper initial stiffness and flowability of materials, but also outstanding thixotropy, which ensures sufficient yield stress self-adjusted by materials within a certain operating time to bear the weight of the whole stacked layers without collapse and deformation. Based on the printable window determined by the initial flow state and RST of the materials, the printable layers of the 10 mixtures in this study were calculated, thus a verification experiment was conducted by using a laboratory printer. Some representative samples are shown in Figure 16. The fresh control mix is in a flow state, which means the extrusion and layers deposition can be completed, as observed in the Figure 16a. The fluidity of the three mortars single-doped with TP is high, so the continuous extrusion of materials through nozzle is fine while the shape retention ability is poor and the bottom layer is seriously deformed during the stacking of layers, as shown in Figure 16b,c which are the experimental printing verifications of TP-1 and TP-2, respectively. In the groups single-doped with NC, the fluidity is low, which is good for stacking performance but not beneficial to the extrusion of 3DPC, as shown in Figure 16d–f for NC-1, NC-2, and NC-3, respectively. The cement mortars mixed with double-doped TP and NC are shown in Figure 16g,h, where M-1 mixture is extruded continuously and additionlly the stacked layers bond tightly without obvious collapse and deformation. This shows the perfect synergy of extrudability and buildability in 3DPC and agrees with the calculated RST results. It can be seen that the 3‰ TP addition promotes the flow state, slump retention and extrudability of the mixture, meanwhile 7‰ NC ensures the structural accumulation performance of the mortar. Therefore, the 3D printing buildability of mortar can be improved by compounding 7‰ NC and 3‰ TP water reducing agent. Moreover, excellent buildability of 3DPC can be obtained by controlling RST and the fluidity of printed mortar to 1.0~2.0 Pa/s and 170~200 mm.

#### 3.4.4. Printable Windows

For 3DPC, the pumpability and extrudability of materials can be investigated by the respective lifetime windows on the initial viscosity-yield stress-fluidity diagrams. The upper and lower limits define the acceptable value settings of these parameters [45]. The buildability of the material can be used to characterize the early structure construction rate of the material through RST. High RST may cause quick flocculation of materials and fast formation of solid microstructures. Then the cement particles enter the structuration process dominated by cement hydration, which is irreversible. As a result, despite any good stacking properties of the materials, they will lose their plasticity quickly, which is harmful to the extrusion of 3DPC. It has been reported in a recent study that both the static yield stress and dynamic yield stress can affect extrusion performance and structural performance of 3DPC [46,47]. In 3DPC, low dynamic yield stress for shearing in nozzle and high static yield stress for stack of layers on the platform are required [48,49]. After the materials are pumped and extruded, the microstructures formed by the intermolecular force of cement particles and CSH bridge are destroyed, and the materials require static yield stress by quick flocculation for rebuilding the microstructure, which means a rapid yield stress transition from dynamic to static is necessary. This requires excellent thixotropy, because of that low change rate of static yield stress will lead to low flocculation rate and low structure rebuilding rate, which will result in the collapse and unability to be piled up of printed materials. Therefore, an appropriate RST is significantly important for the shape retention of the deposited materials, which thus can be regarded as a building block for the build-up ability in 3DPC.

**Figure 17:**
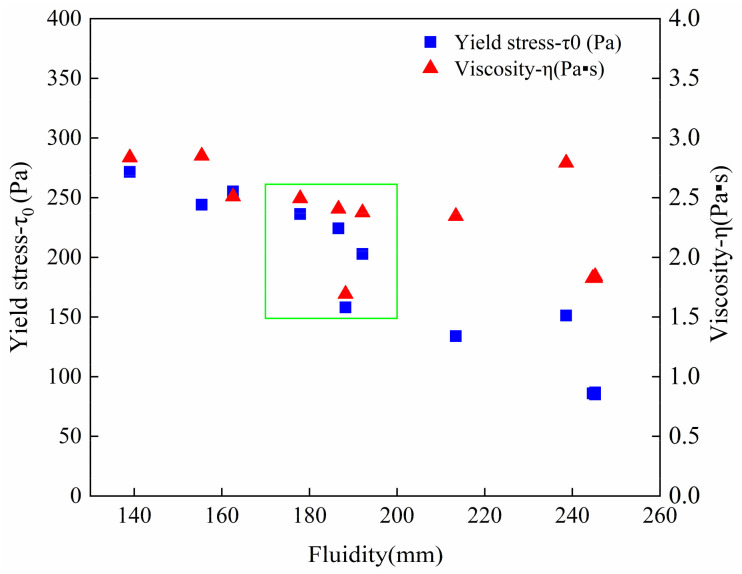
The printable window of yield stress, viscosity and fluidity.

The fluidity test connects the difference in the flow state of mixture with the damage of microstructure during the printing process through the uniform vibration of cement mortar [39]. According to the 3D printing verification of the 10 mixture groups, a printable window is established from the initial viscosity, initial yield stress and fluidity test results through the printing effect. As shown in Figure 17, the parameter dots that can be used for 3DPC are located in a dense area of the dots, which forms a printable window: the ranges of the initial yield stress, initial viscosity and fluidity are 150~250 Pa, 1.6~2.6 Pa·s and 170~200 mm, respectively.

**Figure 18:**
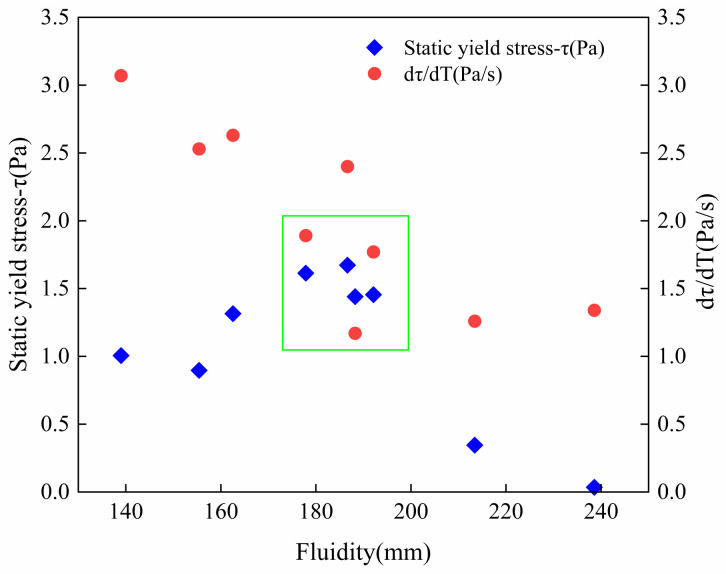
The printable window of static yield stress, RST and fluidity.

According to printing experiments of the 10 sets of samples, the printable interval of initial static yield stress—RST—fluidity is established. As shown in Figure 18, the range of dense points of parameters can be obtained as follows: initial static yield is 1.2~1.7 kPa, RST is 1.0~2.0 Pa/s, the fluidity is 170~200 mm, the recommended dosage of NC is 7‰, and the recommended dosage of TP is 3‰. In addition, the further discussion about the action mechanism of TP and NC on the buildability of 3DPC has been shown in Appendix A.

## 4. Conclusions

The synergistic effect of a self-made ester-ether copolymerization thixotropic superplasticizer (TP) and nano-clay (NC) on the buildability of 3D printable cementitious materials (3DPC) have been investigated in this study. The initial flowability, initial rigid and thixotropic properties are measured by fluidity tests, green strength tests and rheological tests. A rheology model has been used for evaluation of the buildability of fresh 3DPC, and printing tests have been conducted for verification. The following major conclusions are drawn:

(1) A thixotropic superplasticizer (TP) not only improves the initial fluidity of materials but also has a slump retention effect that increases the initial rheological parameters for guaranteeing sufficient initial stiffness of “standable printed materials”. What’s more, nano-clay (NC) improves the growth rate of static yield stress in 3DPC, which ensures the strength development required by the buildability of materials.
(2) Combinations using NC and TP can control the reflocculation rate and structuration rate of cement mortars at the same time. A suitable printing “ink” with excellent extrudability and buildability is obtained when the dosage of NC is 7‰ and the dosage of TP is 3‰ of cementitious materials.
(3) Static yield stress had a good correlation with green strength, which provided a simplified test and evaluation method for the early mechanical properties of 3DPC.
(4) A rheological evaluation method of buildability in 3DPC was proposed: only when the rate of static yield stress evolution over time (RST) of 3DPC is over $\rho gh/\left(\sqrt{3}t\right)$, excellent buildability can be obtained.
(5) A printable window of 3DPC was obtained: the cement mortars with a RST range of 1.0–2.0 Pa/s or fluidity value range of 170~200 mm have better printability.

## Figures and Tables

**Table 1:** Chemical compositions [wt.%] and Blaine fineness of the cement and NC.

Materials	SiO_2_	Al_2_O_3_	Fe_2_O_3_	CaO	MgO	K_2_O	SO_3_	Na_2_O	Ti_2_O	LOI	Blaine Fineness
Cement	23.05	6.45	2.98	60.69	1.48	0.21	2.49	0.15	-	1.02	360 m^2^/kg
NC	58.4	26.73	0.51	9.62	0.20	3.05	-	0.21	0.15	1.13	251 m^2^/kg

**Table 2:** Mix design of 3D printing concrete (kg/m^3^).

Materials	Control	TP-1	TP-2	TP-3	NC-1	NC-2	NC-3	M-1	M-2	M-3
Cement	1000	1000	1000	1000	1000	1000	1000	1000	1000	1000
Sand	1200	1200	1200	1200	1200	1200	1200	1200	1200	1200
Water	320	320	320	320	320	320	320	320	320	320
PCE/‰	3	0	0	0	3	3	3	0	0	0
T-PCE/‰	0	2.5	3	3.5	0	0	0	3	3	3
NC/‰	0	0	0	0	7	8	9	7	8	9

**Table 3:** Comparison of calculated value and measured value of RST in 3DPC.

Layers	Time (s)	Calculated *R_thix_*_,_*_min_* (Pa/s)	Measured *R_thix_* (Pa/s)
1	300	0.75	2.47
2	360		2.10
3	420		1.67
4	480		1.24
5	540		0.83
6	600		0.72

**Figure A1:**
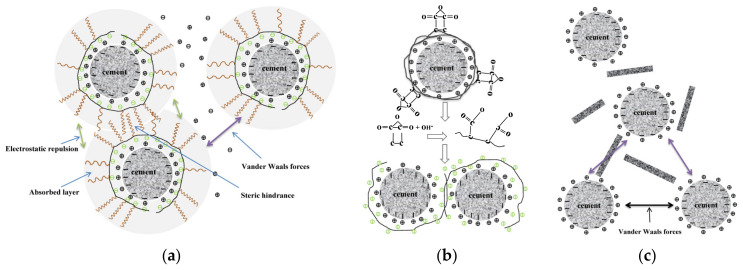
(**a**) The Brownian motion and van der Waals force between cement particles and action mechanism of (**b**) PCE and (**c**) TP on cement particles.

## Data Availability

Data sharing is not applicable.

## Appendix A. Discussion of the Action Mechanism of TP and NC

Cement is a cementitious material with chemical reactivity. After mixing with water, because of the Brownian motion of cement particles and van der Waals force (as shown in Figure A1a, cement particles continuously collide to form a flocculated structure, which significantly reduces the fluidity and plasticity of the mortar and is detrimental to the application of engineering construction). Superplasticizers such as polycarboxylate superplasticizers, naphthalene superplasticizers and aliphatic superplasticizers, etc. are often used to adjust the rheological properties and workability of fresh cement mortar at early age, Polycarboxylate superplasticizers is used as a benchmark in this study. As shown in Figure A1b, each particle is charged with the same charge on the surface as a result of the oriented adsorption of active molecules on cement particles surface. Hence, under the electrostatic repulsion effect, the system is in a relatively stable suspension state and the initial flocculation structure formed after the addition of water is destroyed. Meanwhile, the free water wrapped by cement particles is released and the volume fraction of dispersed phase is reduced, which can effectively improve the rheology of cementitious materials [50]. In addition, hydrophilic branched chains of superplasticizer extend in the aqueous solution, thereby forming a hydrophilic three-dimensional adsorption layer in certain thickness on the adsorbed cement particles surface. When the particles are close to each other, the adsorption layer begins to overlap, which is called steric hindrance effect between cement particles. The more the overlap, the stronger the steric hindrance effect, which result in the hindrance to cement particles flocculation greater. Besides, the addition of superplasticizers will lead to higher surface coverage of the polymer, thicker effective layer and weaker maximum attractive force between particles. Thereby, the number of sites for nucleation is decreased and the bridging distance between particles is increased, which leads to the improvement of 3DPC flowability [51,52]. The contribution of interaction between cement particles surface and initial cement hydration to thixotropy depends on the particle distance. However, the presence of superplasticizers increases the distance between the particles and significantly reduces the flocculation rate of fresh concrete, thus the thixotropy is lowered [53].

The ester-ether copolymerized thixotropic superplasticizer used in this research is different from the polycarboxylic acid-based superplasticizer, as shown in Figure A1c. When TP is added in the cement mortar, a part of the superplasticizer molecules intercalates into the layered hydration products of C_3_A to form a calcium aluminate-thixotropic agent intercalation hydrate, which consumes and adsorbs a part of the water-reducer molecules. The other part of TP molecules is adsorbed on the surface of cement particles, the same as in ordinary polycarboxylate superplasticizer. TP molecules contain polyoxyalkylene groups-(CH_2_CH_2_O)_m_-R, in which the oxygen in the ether bond can form a stable hydrogen bond with water and form a hydrophilic three-dimensional protective film. The protective film can not only plays a role in infiltration, but also provides steric hindrance, which can delay the physical flocculation of cement particles and play a role in adsorption and dispersion. However, unlike ordinary polycarboxylic acid water-reducing agents, the coverage area of TP on the surface of cement particles is not as large as in PCE. What’s more, the dispersion performance of TP on cement particles is not as good as that of PCE, due to the higher content of ester groups in the molecular structure of TP. Compared with the viscosity of mortar added PCE, that of mortar with TP is larger. The particles in cement mortar with TP can form many three-dimensional network structures. These structures can be restored immediately after being broken by a shearing process, which is called excellent thixotropy.

However, along with the cement hydration, on the one hand, the ester groups will slowly be hydrolyzed in the alkaline solution produced by the cement hydration. Then a desorption process occurs, releasing the superplasticizer molecules, which were adsorbed on the cement particles originally without contributions to the dispersibility, slowly into the solution again. The concentration of superplasticizer in the solution is maintained at a certain level, leading to that the dispersibility is maintained for a period of time without loss, which makes great contributions to the maintenance of workability. On the other hand, in the presence of sulfate, sulfate ions can replace the water-reducing agent molecules that have been inserted into the layered hydration products and form AFt or AFm phases with the hydration products of C_3_A. Afterwards, the adsorbed water reducing agent molecules are re-released into the solution, which can act on the cement particles through water reducing and dispersing, so that the cement particles dispersion retention time is prolonged. Acting as a “storage room” of water-reducing agent molecules, TP releases the dispersion groups gradually with the cement hydration, which is different from PCE. PCE molecules are all exposed to the cement suspension system at once the water is added in the mixtures. Therefore, TP can adjust the workability of mortar without significantly weakening its thixotropy. However, TP just partly make up for the adverse effects of PCE on the thixotropy of the mortar, it does not essentially enhance the thixotropy of cement-based materials.

NC is a common thixotropic adjustment admixture in 3DPC. NC addition in the self-compacting concrete can significantly improve concrete thixotropy and reduce the pressure from formwork [54,55]. Adding NC in 3DPC can both effectively improve the early construction rate and buildability of concrete and speed up the vertical printable rate [20,56]. The fibrous structure of NC makes it bundles and interlaced in the cement mortar, thus a disordered grid is formed. The messy grid has a binding effect on the cement mortar and aggregates, which is manifested as an obvious rise in the static yield stress of fresh cement mortar [57]. The particle size distribution of colloidal system will not be changed by NC in the cement mortar, but the bonding between particles/aggregates will be increased [58]. Compared with the soft bonding mechanism of PCE, the addition of NC provides a relatively rigid bond. Therefore, the storage modulus [59] is increased and the critical strain is decreased, which improves the thixotropy of mortar significantly. In addition, NC particles can absorb the moisture in the mortar, so that the volume fraction of the dispersed phase will be decreased for increasing the static yield stress of the slurry [38,46]. Overall, the combined use of thixotropic superplasticizer and NC can both improve and control the rheological and thixotropic properties of 3DPC, so that we can adjust the 3D printing properties for required practical engineering applications.
